# *Plasmodium* *falciparum* Malaria, Southern Algeria, 2007

**DOI:** 10.3201/eid1602.090914

**Published:** 2010-02

**Authors:** Saïd C. Boubidi, Ibrahim Gassen, Yacine Khechache, Karima Lamali, Boualem Tchicha, Cécile Brengues, Michela Menegon, Carlo Severini, Didier Fontenille, Zoubir Harrat

**Affiliations:** Institut Pasteur, Algiers, Algeria (S.C. Boubidi, Z. Harrat); Prevention Centre, Tamanrasset, Algeria (I. Gassen); Institut National de Santé Publique, Algiers (Y. Khechache, B. Tchicha); Ministère de la Santé, Algiers (K. Lamali); Institut de Recherche et de Développement, Montpellier, France (C. Brengues, D. Fontenille); Istituto Superiore di Sanità, Rome, Italy (M. Menegon, C. Severini)

**Keywords:** Anopheles gambiae, malaria, Algeria, Plasmodium falciparum, transportation, antimalarial drug resistance, parasites, climate, dispatch

## Abstract

An outbreak of *Plasmodium falciparum* malaria occurred in Tinzaouatine in southern Algeria in 2007. The likely vector, *Anopheles gambiae* mosquitoes, had not been detected in Algeria. Genes for resistance to chloroquine were detected in the parasite. The outbreak shows the potential for an increase in malaria vectors in Algeria.

Outbreaks of malaria in southern Algeria have been observed for many years, including a major epidemic in Dajnet in 1928–1929 (*1*). Most (>90%) documented cases were attributed to *Plasmodium falciparum* (*2*); *Anopheles sergenti* and *An*. *multicolor* mosquitoes were incriminated as potential vectors (*3*). The Sahara Desert has been regarded as an effective barrier against northward expansion of *An*. *gambiae* mosquitoes, the main malaria vector in Africa, into Algeria. However, this mosquito has been detected near the Algeria–Mali border (*4*).

In recent years, marked changes in the environment and the economy of southern Algeria have occurred (exploitation of underground water resources, growth of the human population in several oases, and development of a transport infrastructure). The new Trans-Saharan Highway, which links Algeria and West Africa, is a potential route for introduction of tropical vectors and parasites into southern Algeria (*2**,**5*).

In November 2007, a total of 26 autochthonous cases of *P*. *falciparum* malaria were detected in Tinzaouatine, a village in Algeria near the Algeria–Mali border. We present results of a parasitologic and entomologic study conducted during the outbreak and discuss the potential for establishment of vectors and *P*. *falciparum* malaria in Algeria.

## The Study

Tinzaouatine (altitude 620 m, 19.95°N, 2.96°E, population ≈12,000) is a village near the Mali border, ≈2,000 km south of Algiers and 578 km southeast of Tamanrasset. Most of its inhabitants are nomadic Tuareg. The climate is arid (annual mean temperature 27°C, range 17°C–33°C, <100 mm rain/year; Office Nationale de Météorologie, Algiers, Algeria). Precipitation is associated with the West African monsoon and restricted to a short period (June–September). The Tinzaouatine River, which is dry for most of the year, occasionally floods. After flooding, receding water results in abundant pools (*gueltas*) that are ideal breeding sites for anopheline mosquitoes. Livestock (mostly sheep and goats) are common in the region. However, no agricultural activity takes place and no irrigation system exists.

During a 2-week period in December 2007, adult mosquitoes were collected by morning indoor spraying in 4 houses and 3 nomad tents (2×/week), human landing catches (2 nights/week), and CDC light traps and mouth aspirators in resting sites (at night). Adult sampling was conducted in dwellings of persons with cases of malaria. Human landing catches were made on 2 adult volunteers from the medical research team from 8:00 pm to 6:00 am. Larvae and pupae were collected by dipping into 2 mosquito-positive pools (1× over a 3-hour period). Adult mosquitoes derived from pupae were identified by using morphologic keys (*6*) and genotyped by rDNA PCR to determine species within the *An*. *gambiae* complex (*7*).

Eleven pools were tested for anopheline larvae. However, many had been treated with insecticide. Larvae of anophelines and other species were collected from 2 gueltas (area 30 m^2^ and 200 m^2^, respectively). A total of 123 anopheline larvae were reared into adults (35 males and 12 females hatched). All specimens were of the Mopti (M) molecular form of *An*. *gambiae* *sensu stricto* mosquitoes. Use of entomologic controls at the same site in 2008 confirmed that the unique anopheline species present in this area was *An*. *gambiae sensu lato*. No adult mosquitoes were captured, probably because of insecticide spraying during the period of sampling to control the outbreak and because of a temperature <10°C at night.

Clinical diagnosis of malaria was made at the health center in the village. A total of 1,468 samples were examined by microscopy during the outbreak. Twenty-six patients (11 female and 15 male, age range 1–43 years) who had fever, chills, and rigor had samples positive for *P*. *falciparum*. None of these patients had traveled outside Tinzaouatine before the outbreak, which indicated that these cases were autochthonous.

All patients were treated with chloroquine (10 mg/kg/day for 2 days and 5 mg/kg for 3 days). Clinical resistance to chloroquine was not reported and no deaths occurred. Informed consent was obtained from each patient or adult guardian of children enrolled in this study at the time of blood collection.

Ten samples were chosen for molecular study; 8 were from patients positive by microscopy and 2 were from patients with malaria symptoms negative for *P*. *falciparum* by microscopy. Samples were processed by placing a drop of blood on filter paper. Molecular analysis was conducted according to the protocol described by Snounou et al. (*8*). Molecular screening by real-time PCR was used to detect mutations in the *P*. *falciparum* dihydrofolate reductase (*dhfr*), dihydropteroate synthase (*dhps*), and chloroquine resistance transporter (*crt*) genes, which are involved in *P*. *falciparum* drug resistance (*9*). Molecular analysis results of 5 PCR-positive blood samples showed the *pfcrt* 76T and the *dhfr* 108N mutations, and 4 showed the quadruple mutation (*dhfr* 51I, 59R, 108N and *dhps* 436A). No mutations were detected in the *dhps* 540 codon. We also identified a *P*. *falciparum* isolate with a unique sextuple drug resistance profile (Y_86mdr1_/T_76crt_/I_51dhfr_/R_59dhfr_/N_108dhfr_/A_436dhps_).

Although all patients were treated with chloroquine and despite our evidence of polymorphisms in genes linked to chloroqunie resistance, no clinical failures were observed. Polymorphisms in the *crt* gene are strongly associated with chloroquine resistance. Involvement of the *mdr1* gene in chloroquine resistance has been challenged, but variation at codon 86 of this gene modulates resistance to chloroquine (*10*).

## Conclusions

An average of 300 cases of malaria is recorded in cities in southern Algeria every year, mostly in Tamanrasset and Adrar. Parasites are introduced by infected humans; >90% of cases originate in Mali and Niger (*11*). Several autochthonous infections have been reported in Tinzaouatine (Table). We suggest that introduction of malaria into this area likely reflects the highly mobile nature of local populations and associated travel to or from areas endemic for malaria. Interethnic conflicts in northern Mali have also increased the displacement of populations toward Algeria.

**Table:** Cases of autochthonous malaria, Tinzaouatine, Algeria, 1999 and 2003–2005*

Species	No. case-patients			
	1999	2003	2004	2005
*P*. *falciparum*	4	11	1	0
*P*. *vivax*	0	1	3	1
*P*. *malariae*	0	0	0	0
*P*. *ovale*	0	0	0	0

*All case-patients were positive for *Plasmodium* spp. by microscopy and treated with chloroquine.

*An*. *gambiae* mosquitoes in Algeria probably originated in Mali. Algeria has borders with Mali and Niger, countries where *An*. *gambiae* *sensu lato* mosquitoes are present (*4*). The most likely mode of introduction of *An*. *gambiae* mosquitoes into Algeria is passive transport by vehicles and trucks because considerable traffic moves across its borders (*12*). An alternative hypothesis is that mosquitoes were carried by wind from breeding sites in southern Mali, a mode of dispersal that has been described for other species of mosquitoes (*13*).

*An*. *gambiae* mosquitoes found in Algeria were of the M molecular form. Current knowledge of genetics and distribution of *An*. *gambiae sensu lato* mosquitoes (*14**,**15*) suggests that this species is probably of the chromosomal M form, which is adapted to an arid climate. Unfortunately, chromosomal inversions in mosquitoes found in Algeria could not be assessed because of lack females at the half-gravid stage (during blood digestion and egg development). However, conditions in 2007 were probably favorable for establishment of this species because the infested area had experienced unusual heavy rainfall, particularly in November a (Figure). Moreover, the location of this species ranges from the Sahel in West Africa to rain forests in equatorial regions (*14*).

**Figure:**
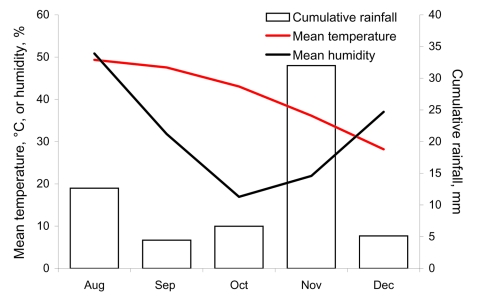
Climatic data for Tinzaouatine, Algeria, August–December 2007 (Algerian Ministry of Meteorology).

We cannot predict whether this highly effective vector will become established in southern Algeria, with an accompanying risk for establishment of malaria, or whether this region will be a source of vectors and parasites that could threaten the rest of Algeria. The bionomics of this species and its ability to survive the long hot and dry season are among several factors that need to be investigated.

## References

[1] Brousses A. Contribution à l’étude du paludisme en région saharienne. Observations recueillies à Djanet au cours de l’épidémie de 1928–1929. Arch Inst Pasteur Alger. 1930;8:77–85.

[2] Benzerroug EH, Janssens PG. Surveillance of malaria in the Algerian Sahara [in French]. Bull Soc Pathol Exot Filiales. 1985;78:859–67. 3836773

[3] Holstein M, Le Corroller Y, Addadi K, Guy Y. The *Anopheles* of the Sahara [in French]. Arch Inst Pasteur Alger. 1970;48:7–15. 5527688

[4] Doumbo O, Toure A, Coulibaly A, Koita O, Traore B, Dolo A, Les aspects parasitologiques de l’épidémiologie du paludisme dans le Sahara Malien. Med Afr Noire. 1991;38:103–8.

[5] Ramsdale CD, de Zulueta J. Anophelism in the Algerian Sahara and some implication of the construction of a trans-Saharian highway. J Trop Med Hyg. 1983;86:51–8. 6887315

[6] Brunhes J, Rhaim A, Geoffroy B, Angel G, Hervy JP. Les culicidae de l’Afrique Méditerranéenne. Un logiciel d’identification et d’enseignement. Montpellier (France): Institut de Recherche et de Développement; 1999.

[7] Fanello C, Santolamazza F, della Torre A. Simultaneous identification of species and molecular forms of the *Anopheles gambiae* complex by PCR-RFLP. Med Vet Entomol. 2002;16:461–4. 10.1046/j.1365-2915.2002.00393.x 12510902

[8] Snounou G, Viriyakosol S, Zhu XP, Jarra W, Pinheiro L, do Rosario VE, High sensitivity of detection of human malaria parasites by the use of nested polymerase chain reaction. Mol Biochem Parasitol. 1993;61:315–20. 10.1016/0166-6851(93)90077-B 8264734

[9] Duraisingh MT, Jones P, Sambou I, von Seidlein L, Pinder M, Warhurst DC. The tyrosine-86 allele of the pfmdr1 gene of *Plasmodium falciparum* is associated with increased sensitivity to the anti-malarials mefloquine and artemisinin. Mol Biochem Parasitol. 2000;108:13–23. 10.1016/S0166-6851(00)00201-2 10802315

[10] Babiker HA, Pringle SJ, Abdel-Muhsin A, Mackinnon M, Hunt P, Walliker D. High-level chloroquine resistance in Sudanese isolates of *Plasmodium falciparum* is associated with mutations in the chloroquine resistance transporter gene *pfcrt* and the multidrug resistance gene *pfmdr1.* J Infect Dis. 2001;183:1535–8. 10.1086/320195 11319692

[11] Ministère de la Santé Publique. Rapports annuels du service du paludisme et des maladies parasitaires, Institut National de Santé Publique, 1985–2007. Algiers (Algeria): Le Ministère; 2008.

[12] Chauvet G, Hassani NT, Izri MA. Malarial problems and the trans-Saharan highway [in French]. Bull Soc Pathol Exot Filiales. 1985;78:852–8. 3836772

[13] Service MW. Mosquito (Diptera: Culicidae) dispersal: the long and short of it. J Med Entomol. 1997;34:579–88. 9439109 10.1093/jmedent/34.6.579

[14] Della Torre A, Tu Z, Petrarca V. On the distribution and genetic differentiation of *Anopheles gambiae* s.s. molecular forms. Insect Biochem Mol Biol. 2005;35:755–69. 10.1016/j.ibmb.2005.02.006 15894192

[15] Sogoba N, Vounatsou P, Bagayoko MM, Doumbia S, Dolo G, Gosoniu L, Spatial distribution of the chromosomal forms of *Anopheles gambiae* in Mali. Malar J. 2008;7:205. 10.1186/1475-2875-7-205 18847463 PMC2579919

